# The Performance of Carbonation-Cured Concrete

**DOI:** 10.3390/ma12223729

**Published:** 2019-11-12

**Authors:** Zhen Li, Zhen He, Xiaorun Chen

**Affiliations:** 1 School of Urban Construction, Yangtze University, No.1 Nanhuan Road, Jingzhou 434023, China; lizhen@yangtzeu.edu.cn; 2 State Key Laboratory of Water Resources and Hydropower Engineering Science, Wuhan University, Wuhan 430072, China; xiaorun-123@163.com; 3 China Construction Ready Mixed Concrete Co., Ltd, Fozuling 1st Road, Jiangxia District, Wuhan 430200, China

**Keywords:** carbonation-cured concrete, CO_2_ uptake, pH, chloride ion permeability, abrasion resistance

## Abstract

The research shows that carbonation-cured concrete has several mechanical and durability properties that are better than those of moisture-cured concrete. However, many properties of carbonation-cured concrete have not yet been studied. In this research, carbonation-cured concrete was prepared by pre-curing, carbonation curing, and then moisture curing. The compressive strength, CO_2_ uptake, pH value, chloride ion permeability and abrasion resistance of the carbonation-cured concrete were investigated. Results showed that the compressive strength of carbonation-cured concrete was more than 10% higher than that of moisture-cured concrete at the same age; a steel bar is stable in carbonation-cured concrete; and carbonation-cured concrete exhibited better abrasion resistance and chloride ion permeability than that of moisture-cured concrete. The optimization of pore structure and improvement in the micro-hardness are the reasons for the improved chloride ion permeability and abrasion resistance of carbonation-cured concrete.

## 1. Introduction

In recent years, various forms of precast concrete have been used, for example as components in prefabricated buildings [1] and urban utility tunnels [2], segments in tunnel engineering [3], and sleepers in high-speed railway construction [4,5]. Carbon emissions in the precast concrete industry are high due to the large amount of emissions created by cement production and the high energy consumption of curing methods such as steam curing [6,7,8]. In a low-carbon economy environment, the precast concrete industry develop in the direction of low energy consumption and greening.

Carbonation curing is a new method of curing that is different from the natural carbonation of mature concrete. As the main hydration product in mature concrete, calcium-silicate-hydrate (C-S-H), reacts with atmospheric CO_2_, eventually becoming a silica gel and losing its binding capability in the process of natural carbonation. However, carbonation curing of concrete means that the concrete is cured in a high-pressure atmosphere of pure CO_2_, and the unhydrated cement minerals react with CO_2_ to solidify it in concrete in the form of carbonate. Hence, the hardening process of concrete is accelerated and its mechanical and durability properties are improved [9,10,11,12,13,14,15]. When carbonation curing technology is applied in precast concrete, it not only reduces the carbon emissions of precast concrete products, but also improves the performance of precast concrete.

It is well known that the carbonation reaction lowers the pH value of concrete and may cause corrosion of steel [16]. In order to ensure the stability of steel bar in carbonation-cured concrete, it is necessary to study the effect of carbonation curing on the pH value of concrete. Chloride ion is one of the main causes of steel corrosion in concrete. It destroys the passivation film of the steel bar, forms corrosion microcells, accelerates the anode reaction, and thus causes the corrosion of the steel bar in the concrete [17,18], therefore, the anti-chloride ion permeability of carbonation-cured concrete is worthy of attention. Although the pH value and chloride ion permeability of mature concrete after natural carbonation (by the acceleration method) have been studied [19,20,21], there are few studies on the relevant properties of carbonation-cured concrete. Water conveyance tunnels and large-flow sewage concrete pipelines may cause abrasion damage to concrete that is subject to long-term action of water containing suspended sediment and bed load [22,23], therefore, it is necessary to investigate the abrasion resistance of carbonation-cured concrete.

This study aimed to examine the above problems, by investigating the pH value, chloride ion permeability and abrasion resistance of carbonation-cured concrete. By comparing the differences in pore structure and micro-hardness between carbonation-cured and moisture-cured cement paste, the enhancement mechanism of anti-chloride ion penetration and the abrasion resistance of carbonation-cured concrete was explained.

## 2. Materials and Methods 

### 2.1. Preparation of Samples

The cement used in this study was ordinary Portland cement (OPC), and its chemical composition is shown in Table 1. The coarse aggregate was granite gravel with a maximum particle size of 20 mm. The fine aggregate was river sand with a fineness modulus of 2.9. The water absorption at saturated surface-dry basis of the coarse aggregate and the fine aggregate were 0.31% and 0.93%, respectively. The mixing water was tap water.

**Figure 1:**
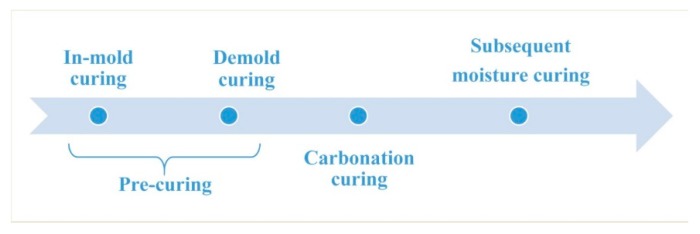
Curing procedure of carbonation-cured concrete.

The water-cement ratio is a key factor affecting the workability and carbonation of concrete. When the water-cement ratio is relatively high, although the concrete has good fluidity, excessive free water will hinder the diffusion of CO_2_ in the concrete and reduce the degree of carbonization [24,25]. When the water-cement ratio is relatively low, although the degree of carbonization is high, the fluidity of the concrete is poor. Therefore, in order to ensure the fluidity of the concrete, the water-cement ratio of concrete in this study was 0.35, and a polycarboxylic acid water reducing agent was used to improve the fluidity of the concrete. At the same time, in order to improve the degree of carbonization of concrete, pre-conservation was used to reduce free water in the sample before carbonation curing [26,27]. The mix proportions of the concrete are shown in Table 2. The stirred mixture was placed in a cubic triple die of 100 mm × 100 mm × 100 mm and shaken on a vibrating table for 30 s. The formed concrete was cured as shown in Figure 1. The specific curing conditions and time of different curing stages are shown in Table 3. The first stage was in-mold curing. After the sample was poured, the mold was cured for 6 h under a temperature of 20 ± 2 °C and relative humidity ≤ 60%. The second step was mold release curing. The sample was demolded, and in the same environment as the first step, air was blown for 6 h at a speed of 1 ± 0.2 m/s, and during this time the sample was turned over to ensure the uniformity of water dispersion loss. After pre-curing, the sample lost about 40% of its water, and the effective water-cement ratio in the sample was about 0.2, and this water-cement ratio was in the range for the best carbonization. The third step was carbonation curing. The sample was cured in a pressure vessel at a pressure of 4 bar of CO_2_ with a purity of 99.9% for 12 h. The fourth step was conventional curing. The carbonation-cured sample was cured for 6 days or 27 days in a curing box with a temperature of 20 ± 2 °C and humidity of ≥ 95%. The carbonation-cured concrete on the 1st, 7th, and 28th day of aging were named C1d, C7d, and C28d, respectively. Moisture-cured concrete was used as reference, and the curing conditions were: temperature 20 ± 2 °C, RH ≤ 95%. They were named H1d, H7d and H28d according to their age.

In order to study the mechanism for enhancing the strength and durability of carbonation-cured concrete, paste samples were also formed. The sample size was 40 mm × 40 mm × 40 mm. The water-cement ratio of the paste sample was 0.35. The curing method of the paste samples was consistent with that of the concrete samples.

### 2.2. Testing the Compressive Strength

The samples that were cured at a corresponding age were tested for compressive strength, which was taken as the average value of three test pieces. The strength values were tested using a WAY-2000 electro-hydraulic material testing machine (Xiyi Construction Machine Factory, Wuxi, China).

### 2.3. Methods Used to Calculate Carbon Sequestration

The carbon sequestration of carbonation-cured concrete was calculated using two methods. The first method calculated the carbon sequestration by the mass change in the carbonation-cured concrete, as shown in Equation (1):

CO_2_ uptake (%) = (m*_final_* − m*_innitial_* + m*_evaporated_*) × 100%/m*_cement in sample_*, (1)

where m*_final_* is the mass of concrete after carbonation curing, m*_initial_* is the mass of concrete before carbonation curing and after initial curing, m*_evaporated_* is the water evaporated during the carbonation curing process, which was collected on the walls of the pressure vessel through the absorbent paper after the carbonation curing was completed, and m*_cement in sample_* is the mass of cement in the sample; the above parameters were in units of g.

The second method calculated the carbon sequestration by thermal analysis. Due to the different degrees of carbonization at different depths of the sample, 50 g of mortar samples without coarse aggregate was taken from each of the five layers of the samples: 0–10 mm, 10–20 mm, 20–30 mm, 30–40 mm, 40–50 mm. The mortar sample with a total mass of 250 g was ground and heated consecutively to 105 °C, 500 °C and 1000 °C. After heating, the sample was taken out and cooled to room temperature. After the sample was weighed, it was then heated in a high temperature furnace to the next target temperature. The difference between the mass of the sample at 500 °C and the mass at 1000 °C was due to the decomposition of CaCO_3_ to produce CO_2_. Therefore, the equation for calculating the carbon sequestration of the sample is as shown in Equation (2):

CO_2_ uptake (%) = (sample weight at 500 °C − sample weight at 1000 °C) × 100%/sample weight at 105 °C × 0.43, (2)

where 0.43 is the binder-sand ratio of the sample. The high temperature furnace was a SX2-12-16A energy-saving high temperature box type resistance furnace (Yingshanjianli Electric Furnace Manufacturing Co., Ltd, Huanggang, China) with a silicon molybdenum rod as the heating element, a rated voltage of 380 V, rated power of 12 kW and a maximum operating temperature of 1600 °C.

### 2.4. Testing the pH

Two methods were used to test the pH of the concrete section after splitting. The first method used ethanol phenolphthalein solution to spray the surface of the cracked concrete. When the pH was lower than 9.5, the concrete surface did not change color, and when the pH value was greater than 9.5, the concrete surface turned purple. The second method used the PH110 (Extech, Nashua, NH, US) with a flat head sensor to test the pH. After the cube test piece was split, a piece of 10 mm × 10 mm absorbent paper was put on the point where the pH was to be measured, and then 100 μL of distilled water was dripped onto the absorbent paper. The absorbent paper was closely attached to the concrete section. After 15 min, the distilled water and the concrete pore solution were fully ion exchanged to reach equilibrium. The pH value of the absorbent paper was measured as the pH of the measuring point of the concrete [26,28].

### 2.5. The Electric Flux Test Method for Concrete 

Electric flux is an indicator for evaluating the corrosion resistance of concrete against chloride ions and can reflect the compactness and durability of concrete to some extent [29,30]. In order to compare the differences in resistance to chloride ion penetration and compactness between the carbonation cured concrete and moisture-cured concrete, electrical flux tests were performed on concrete samples at 28 days. The electric flux tester used in the test was the CABR-RCMP6 concrete chloride ion diffusion coefficient and electric flux meter (Jianyanhuace Instrument & Equipment Co., Ltd., Beijing, China).

### 2.6. Method for Testing the Abrasion Resistance of Concrete 

**Figure 2:**
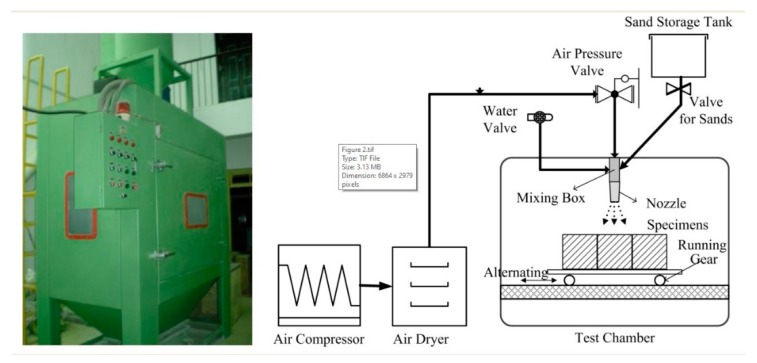
Abrasion test device and its schematic diagram.

The abrasion resistance of concrete samples was tested in a self-designed experimental device [22,23]. The schematic is shown in Figure 2. In this device, the rate of erosion can be varied by adjusting the pressure of the compressed gas. The angle of impact can be changed by a variable angle stage. The specimen can be reciprocated via running gear in the test chamber. Through the programmable control system, parameters such as the abrading time and the number of cycles can be customized. In addition, the device was also equipped with a dust collection system to ensure that it meets environmental requirements.

In order to compare the difference in abrasion resistance between carbonation-cured concrete and moisture-cured concrete, samples at 28 days were selected for abrasion resistance testing. The specific parameters of the test are shown in Table 4. The loss in the sample weight after each shot and the surface topography after the grinding were recorded.

### 2.7. Method to Test the Pore Size Distribution

A sample of the pulverized pulp with a volume of about 10 mm × 10 mm × 10 mm was taken and dried in a vacuum drying oven at a temperature of 65 °C for more than 48 h for the pore structure test. The pore structure was tested using a Autopore 9620 mercury intrusion meter (Micromeritics, Norcross, GA, US). The mercury intrusion analyzer had a maximum pressure of 25 psia for low pressure analysis with a low pressure pore size analysis range of 360–3.6 μm. The mercury intrusion analyzer also had a maximum pressure of 60000 psia for high pressure analysis with a high-pressure pore size analysis range of ≥ 3 nm, and a volumetric accuracy of 0.1 μL for entering mercury and withdrawing mercury.

### 2.8. Test Method for Micro-Hardness 

A 10 mm × 10 mm ×10 mm sample was cut from the inside of the paste block and polished stepwise using a EcoMet 300 polisher (Buehler, Esslingen am Neckar, Germany). After polishing, it was placed in absolute ethanol to terminate hydration for more than 48 h. Then the sample was put in a vacuum oven and kept at 65 °C for more than 48 h. The micro-hardness of the sample was measured using a HXS-1000A digital intelligent micro-hardness tester (Shangguang New Optical Technology Co., Ltd, Shanghai, China). The test load can be set from 10 to 1000 gram of force, the force retention time range is 1–99 s, the objective magnification for measurement is 40 times, and the magnification of the eyepiece is 10 times. The load and load time were set to be 100 gram of force and 10 s, respectively. 

## 3. Results and Discussion

### 3.1. Compressive Strength 

**Figure 3:**
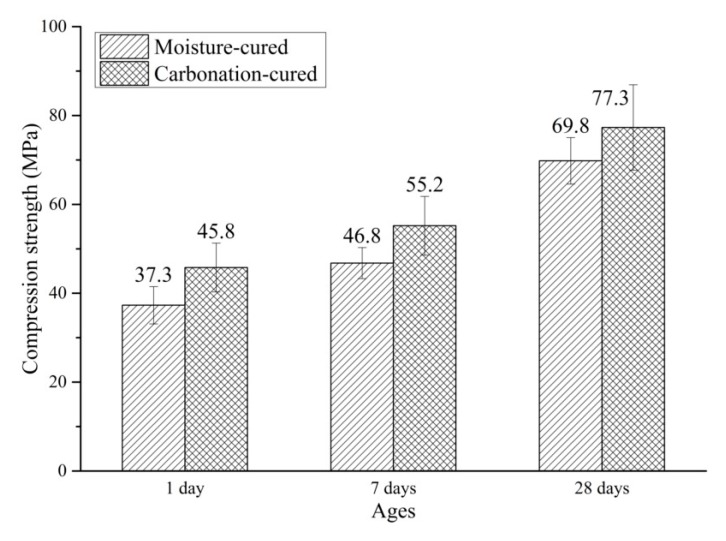
Compressive strength of carbonation-cured and moisture-cured samples.

Figure 3 shows the compressive strength of carbonation-cured concrete and moisture-cured concrete. It can be seen from Figure 3 that after 12 h of pre-treatment and 12 h of carbonation curing, the compressive strength of C1d reached 45.8 MPa. The compressive strength of H1d under conventional curing for 1 day was 37.3 MPa. At 1 day, the compressive strength of carbonation-cured concrete increased by more than 20% compared to that of the moisture-cured concrete. Comparing the compressive strength of C7d with H7d, the strength of C7d was 17% higher than that of H7d. Comparing the compressive strength of C7d with C1d, it can be seen that after the subsequent conventional curing, the compressive strength of carbonation-cured concrete continues to increase, and is still greater than that of the moisture-cured samples at the same age. This indicates that carbonation curing does not hinder and affect the post-hydration of concrete, so that the compressive strength of concrete after carbonation curing continues to increase under subsequent conventional curing conditions. The compressive strength of C28d reached 87.3 MPa, which is more than 10% higher than that of H28d at the same age. It can be seen from the above results that the compressive strength of the carbonation-cured concrete was greater than that of the moisture-cured concrete at the same age, and the increase in compressive strength decreases with age.

### 3.2. CO_2_ Uptake

The CO_2_ uptake of carbonation-cured concrete is shown in Table 5. It can be seen from Table 6 that after 12 h of carbon curing, the CO_2_ uptake is 14–15%. The data measured by the mass change method differs from the data measured by the thermal analysis. Evaporation of moisture in the mass change method may not be fully collected, resulting in data that may be less than the actual value. According to the above pH data, the degree of carbonization in different parts of the carbonation-cured concrete is different. Therefore, when the CO_2_ uptake of the concrete is calculated by the thermal analysis method, samples from the surface to the internal five layers are taken to ensure the accuracy of the calculation. The amount of CO_2_ uptake calculated by the thermal analysis method is slightly larger than that calculated by the mass change method. This is because the CO_2_ decomposed by the carbonate mineral in the cement is also included in the CO_2_ uptake amount, resulting in a value that is higher than the actual value. The CO_2_ uptake of the carbonation-cured concrete at 28 days obtained by thermal analysis was 15.8%, which is slightly higher than the 15.1% at 1 day. This is because the sample also undergoes a carbonization reaction in the process of conventional curing, hence, the amount of CO_2_ uptake increases.

### 3.3. pH Value

**Figure 4:**
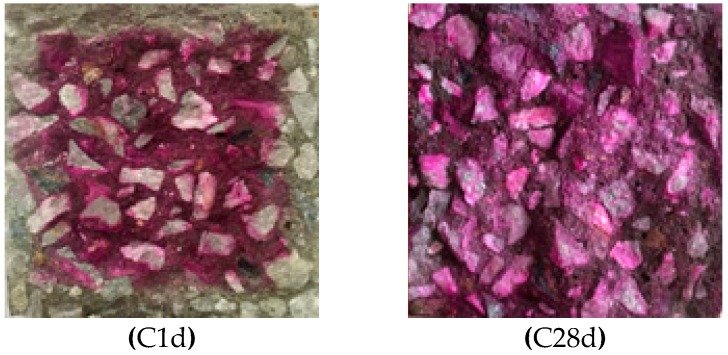
Photos of carbonation-cured concrete C1d and C28d sprayed with ethanol phenolphthalein solution.

Figure 4 shows the sections of C1d and C28d sprayed with phenolphthalein ethanol solution. It can be seen from Figure 4 that after 12 h of carbonation curing, the pH value of the surface of the concrete within 10 mm is less than 9.5 and no discoloration reaction occurred. In the central region of the concrete, the pH is greater than 9.5 and the sample has a purplish red color. From the color reaction of C28d, it can be seen that the entire section of the sample shows a purple-red color, which indicates that after 27 days of subsequent conventional curing, the pH of the entire section of the carbonation-cured concrete is above 9.5.

Table 6 shows the pH values of different parts of the concrete section measured using a pH meter. It can be seen from Table 5 that at 1 day, that is, immediately after the completion of carbonation curing, the pH of the concrete surface layer is less than 9.5, so no phenolphthalein color reaction is observed. In the range of 10–20 mm, although there is a color reaction, the pH is still less than the pH in the 20–50 mm range, which is the innermost layer. From the pH point of view, carbonization occurred in the range of 0–10 mm and 10–20 mm, and a slight carbonization occurred in 20–30 mm range. There was almost no carbonization reaction in the range of 30–40 and 40–50 mm. This indicates that the carbonization reaction occurred only in the depth range of 0–30 mm of the sample, and no carbonization reaction occurred in the interior of the concrete. The reason may be that the pore water in the 0–30 mm depth range of the pre-conservation concrete evaporates to form a channel for CO_2_ diffusion, so the carbonization reaction takes place within this range. However, due to the hindrance of moisture in the pores, CO_2_ failed to reach the range of 30–50 mm, so a carbonization reaction did not occur in the core region of the concrete test block. It can also be seen from the data in Table 5 that the pH value at the surface of the carbonized sample increases to above 12.0 after the subsequent 27 days of conventional curing. This is mainly because carbonization of the cement does not occur in the subsequent moisture-curing process and more Ca(OH)_2_ with a high pH value is produced, so the pH value of the sample increases. The pH value of each layer of the carbonation sample was greater than 12.0.

### 3.4. Electric Flux

**Figure 5:**
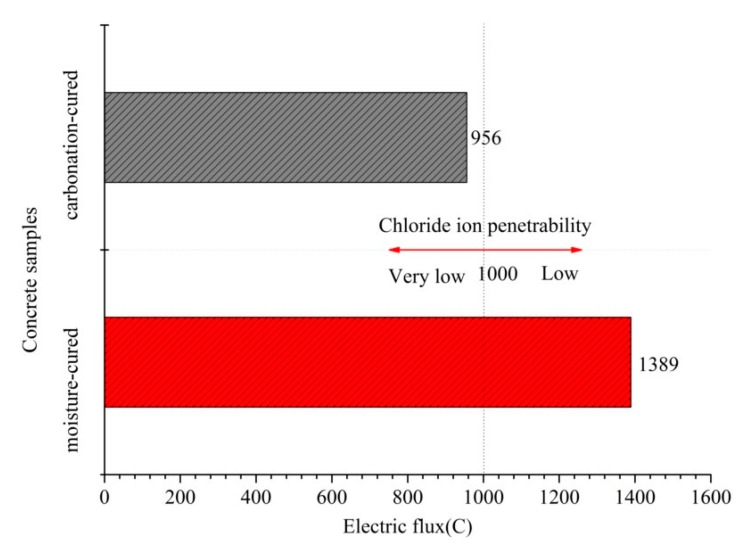
Electric flux of carbonation and hydration curing concrete at 28 days.

Figure 5 shows the electric flux of the carbonation-cured and moisture-cured concrete. It can be seen from Figure 5 that the electric flux value of the carbonation-cured concrete is 956 C which is less than the 1389 C of the moisture-cured concrete. These results indicate that the anti-chloride ion permeability of the carbonation-cured concrete is superior to that of moisture-cured concrete. According to the ASTM C1202 standard for evaluating the chloride ion permeability of concrete [29], the chloride ion permeability of carbonation-cured concrete is characterized as very low.

### 3.5. Abrasion Resistance

**Figure 6:**
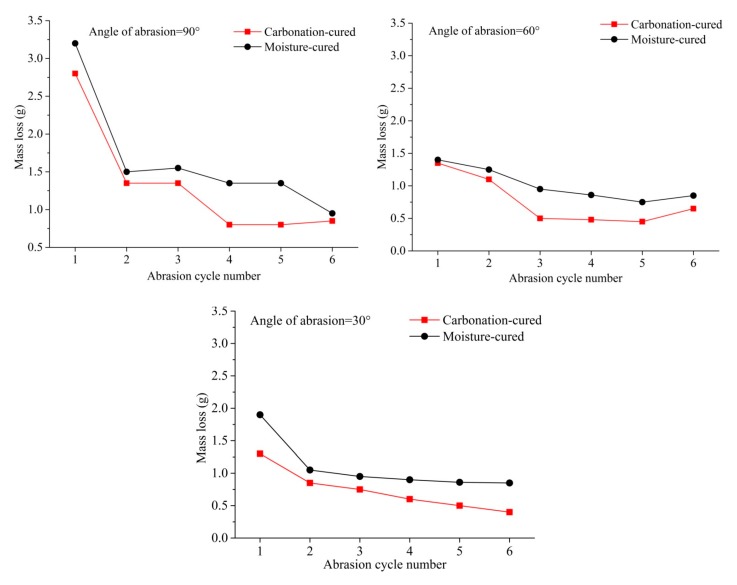
Mass loss vs. abrasion cycle number, (**a**) Angle of abrasion = 90°, (**b**) Angle of abrasion = 60°, (**c**) Angle of abrasion = 30°.

The variation in the mass loss of concrete with the number of abrasion cycle under different angles is shown in Figure 6. It can be seen from Figure 6 that the mass loss of the concrete at the first abrading time is the largest regardless of the angle of abrasion. This is mainly due to a layer of paste on the surface of the sample, which is easily detached from the surface under the impact and cutting of the sand. In addition, it can be seen from Figure 6 that the mass loss of the carbonation-cured concrete is smaller than that of moisture-cured concrete at different angles and different cycles. Studies have shown that when the aggregate of concrete is the same, the abrasion resistance of concrete increases with the increase in compressive strength. Therefore, the results of the abrasion resistance are consistent with the laws of the compressive strength.

**Figure 7:**
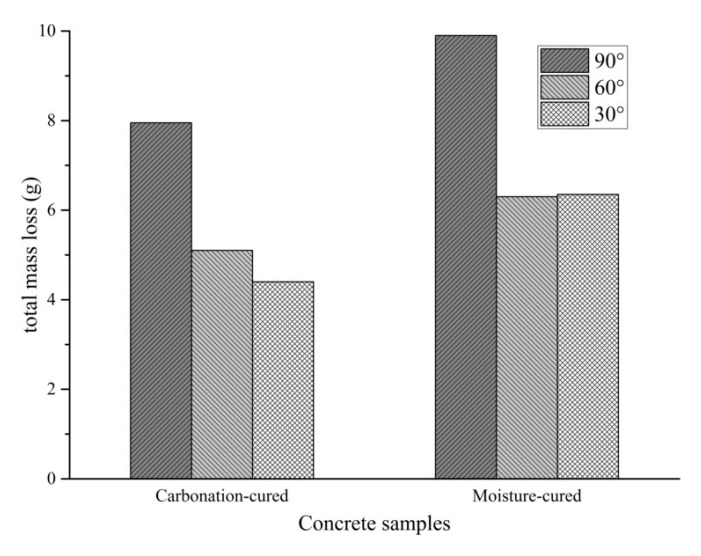
Total mass loss after six cycles of abrasion.

The total mass loss after six cycles of abrading of the sample is shown in Figure 7. According to abrasion erosion theory, the stable mass loss rate of brittle material is largest when the angle of attack of the erosion medium is close to 90°, while the maximum value of the stable mass loss rate of plastic material occurs when the angle of attack is 20°–30°. Therefore, when the abrading angle of the concrete is 90°, the mass loss is significantly greater than 60° and 30°. At the angle of 90°, the effect of sand on concrete is impact damage. Figure 7 shows that the total mass loss of carbonation-cured concrete at the angle of 90° is smaller than that of the moisture-cured concrete. This indicates that the carbonation-cured concrete has better abrasion resistance than conventionally-cured concrete. Considering the difference in the mass loss at the abrading angles of 60° and 30°, there is almost no difference in the total mass loss in the moisture-cured concrete at the two abrasion angles, while for the carbonation-cured concrete, the mass loss at the abrasion angle of 30° is less that at the abrasion angle of 60°. At the abrasion angle of 30°, the effect of the abrasive sand on the concrete surface is mainly cutting, which indicates that the carbonation-cured concrete has better cutting resistance than moisture-cured concrete.

**Figure 8:**
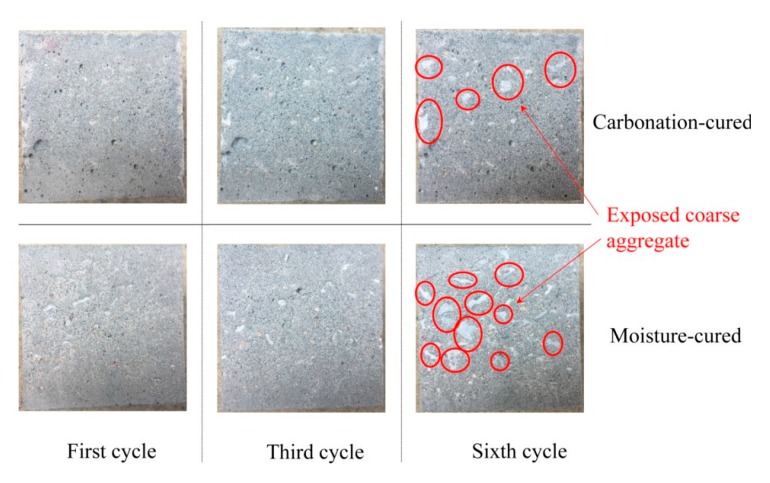
Surface morphology of the concrete samples after different abrasion cycles.

Figure 8 shows the surface morphology of the concrete after different cycles of abrasion angle at 90°. It can be seen from Figure 8 that after the abrading, the floating layer of the concrete is washed away and the mortar leaks. After three cycles of abrading, aggregates appears in the concrete under both curing methods, but the difference is small. However, it is apparent from the surface morphology after six cycles of abrading that the moisture-cured concrete has more exposed aggregates than the carbonation-cured concrete. This indicates that the carbonation-cured concrete has better abrasion resistance than moisture-cured concrete.

### 3.6. Pore Size Distribution

**Figure 9:**
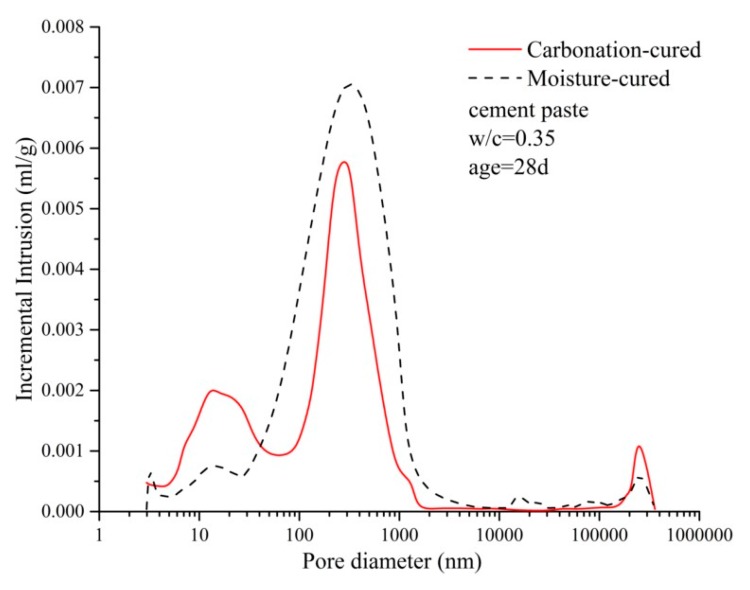
Distribution curve of the pore diameter of carbonation and hydration paste.

Figure 9 shows the pore size distribution curves of the carbonized and hydrated paste samples and there is a clear difference between the two curves. The carbonation-cured paste has a distinct peak at around 10 nm, while the moisture-cured paste has a weak peak here. At 100–1000 nm, the peak area of the moisture-cured paste is significantly larger than that of the carbonation-cured concrete. The integrated area of the curve shows that the total porosity of the moisture-cured paste is greater than that of the carbonation-cured concrete. According to the pore size, the pores are divided into the following ranges: pores below 20 nm are harmless pores; pores of 20–50 nm are less harmful pores; pores of 50–100 nm are harmful pores; and pores above 100 nm are more harmful pores [31,32]. According to the mercury intrusion test data, the pore structure parameters of the carbonation-cured paste and moisture-cured paste were counted and are shown in Table 7. Table 7 shows that the ratio of harmless pores and less harmful pores of the paste is improved after carbonation curing, while the proportion of harmful pores decreases. This is consistent with the test results for macro strength and impermeability. The decrease in the proportion of harmful pores reduces the macroscopic defects of the paste, so as to improve the strength of the concrete [33,34,35]. At the same time, the decrease in the proportion of the more harmful and the harmful pores reduces the proportion of connected pores inside the paste, so as to improve the impermeability of concrete [36,37].

### 3.7. Micro-Hardness

**Figure 10:**
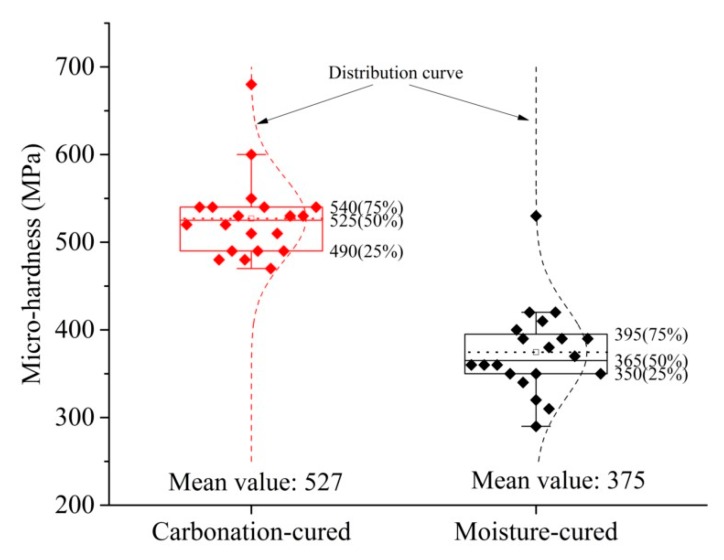
Micro-hardness of carbonation and hydration paste.

Since the data for micro-hardness is highly discrete, the data was processed using a box plot, and the processed data is shown in Figure 10. Outliers can be removed by box plots, making data analysis more reliable. Figure 10 shows that the quartile distance of the two sets of data is small, indicating that the data error is small. Therefore, the average value of the sample is taken as the micro-hardness value of the paste. The micro-hardness of the carbonation-cured paste is 527 MPa, which is 40% greater than that of the moisture-cured paste, which is 375 MPa. The higher the hardness value of the paste, the better the abrasion resistance [22,23,38,39], so the results for micro-hardness explain why the abrasion resistance of carbonation-cured concrete is higher than that of moisture-cured.

## 4. Conclusions

In this study, carbonation-cured concrete was prepared by pre-curing, carbonation curing and then moisture curing. The strength, pH value, CO_2_ uptake, chloride ion permeability and abrasion resistance of the carbonation-cured concrete were studied. By comparing these characteristics in carbonation-cured concrete and moisture-cured concrete, and analyzing the pore structure and micro-hardness of the concrete paste, we were able to make the following conclusions:

(1) The strength of carbonation-cured concrete is more than 10% higher than that of moisture-cured concrete at the same age.

(2) The steel bar in carbonation-cured concrete can exist stably. However, considering the low pH value in the surface layer at the initial stage of carbonation curing, the thickness of the steel protective layer of carbonation-cured concrete should be deeper than that of moisture-cured concrete.

(3) Carbonation-cured concrete exhibited better abrasion resistance than that of moisture-cured concrete.

(4) Carbonation-cured concrete had better chloride ion permeability than moisture-cured concrete.

(5) The pore structure was optimized after carbonation curing, thereby improving the strength and impermeability of the cement paste to some extent.

(6) The abrasion resistance of carbonation-cured concrete was better than that of the moisture-cured concrete because it has greater micro-hardness and compressive strength.

## Figures and Tables

**Table 1:** The chemical composition of the cement.

Sample	Chemical Compositions (%)									
	CaO	SiO_2_	Al_2_O_3_	Fe_2_O_3_	MgO	Na_2_O	K_2_O	SO_3_	CO_2_	LOI
OPC	59.26	22.38	5.73	3.00	2.77	0.28	1.01	3.20	1.45	4.05

**Table 2:** The mix proportions of the concrete.

Cement (kg/m^3^)	Water (kg/m^3^)	Sand (kg/m^3^)	Aggregate (kg/m^3^)	Superplasticizer (kg/m^3^)	*w*/*b*	Slump (mm)
450	158	593	1261	1.808	0.35	154

**Table 3:** Specific curing conditions and time of carbonation curing at different stages.

Curing Period	Curing Conditions
In-mold curing	20 ± 2 °C, RH ≤ 60%, 6 h.
Demold curing	20 ± 2 °C, RH ≤ 60%, blow by wind at speed of 1 ± 0.2 m/s, 6 h.
Carbonation curing	99.9% purity of CO_2_, 4 bar CO_2_ pressure, 12 h.
Subsequent moisture curing	20 ± 2 °C, RH ≥ 95%, 27 days.

**Table 4:** Experimental parameters of abrasion test.

Samples	Angle of Abrasion	Pressure of Abrasion	Cycle Number
C28d	30°, 60°, 90°	6 bar	6
H28d			

**Table 5:** CO_2_ uptake of carbonation curing concrete.

Age of Carbonation-Cured Concrete	CO_2_ Uptake (%)	
	Mass Method	Thermal Analysis Method
1 days	14.2	15.1
28 days	—	15.8

**Table 6:** pH value of concrete samples.

Concrete Samples	Age	pH Value in Different Regions				
		0–10 mm	10–20 mm	20–30 mm	30–40 mm	40–50 mm
Carbonation-cured	1 d	9.0–9.3	9.6–10.2	11.1–13.0	12.8–13.1	12.9–13.0
	28 d	12.1–12.6	12.5–12.8	12.5–12.8	12.9–13.1	12.9–13.1
Moisture-cured	1 d	12.5–12.8	12.6–12.9	12.8–13.1	12.9–13.0	12.9–13.0
	28 d	12.6–12.9	12.8–13.0	12.9–13.0	12.9–13.1	12.9–13.1

**Table 7:** Pore structure parameters of carbonized and hydrated pastes.

Cement Paste	Pore Size Distribution (%)			
	<20 nm	20–50 nm	50–100 nm	>100 nm
Carbonation-cured	17.48	9.82	4.76	67.94
Moisture-cured	10.66	6.23	11.15	72.02
